# Moyamoya Syndrome Presenting as Refractory Status Epilepticus in a 32-Year-Old Female

**DOI:** 10.7759/cureus.13624

**Published:** 2021-03-01

**Authors:** Ali T Alhashem, Khalid H AlSalem, Sarah J Sabihah, Dunya Alfaraj

**Affiliations:** 1 Emergency Medicine, King Faisal University, Al Hofuf, SAU; 2 Emergency Medicine, King Hamad University Hospital, Dammam, SAU; 3 Emergency Medicine, Najran University, Dammam, SAU; 4 Emergency Medicine, King Fahad University Hospital, Imam Abdulrahman Bin Faisal University, Dammam, SAU

**Keywords:** sickle cell disease: scd, moyamoya disease, tramadol, refractory status epilepticus

## Abstract

Moyamoya disease (MMD) is a rare cerebrovascular occlusion condition characterized by ‎progressive stenosis in the terminal portion of the internal carotid arteries on both sides. The measured incidence of MMD is 0.086 per 100,000 people. MMD has variable ‎neurological manifestations, however, seizure is a significant manifestation of MMD with ‎few reported studies. The combination of sickle cell disease (SCD) and MMD confers a much ‎higher risk of ischemic stroke. In this paper, we describe a 32-year-old female with a known ‎case of SCD taking tramadol for a vaso-occlusive crisis, who was presented to the emergency ‎department by a family member due to a low level of consciousness. Despite ongoing ‎management, the patient developed multiple seizure attacks and intubation was ‎performed. A computed tomography (CT) brain angiogram was performed, and the ‎diagnosis of MMD was made. The patient was shifted to the intensive care unit (ICU) and in spite of the ongoing management in the ICU, the patient died. In this case, we highlight the importance of considering MMD as a ‎differential diagnosis when dealing with an SCD patient who developed refractory status epilepticus.‎

## Introduction

Moyamoya disease (MMD) is a cerebrovascular occlusion condition characterized by ‎progressive stenosis in the terminal portion of the internal carotid arteries on both sides, ‎thereby leading to the activation of the collateral vessel network at the base of the brain to ‎compensate for the occlusion ‎[1,2].‎ MMD is rare, with a measured incidence of 0.086 per ‎‎100,000 people. Although MMD primarily affects Asian people, it is found globally in ‎individuals with many ethnic backgrounds ‎[2].‎ It is more prevalent in women than men, ‎with a 2:1 ratio ‎[1].‎ ‎

Although MMD has different neurological manifestations, the most common presentations ‎are ischemia (transient ischemic attacks, stroke, or seizure) and intracranial hemorrhage ‎[2].‎ MMD is reported to be usually associated with sickle cell disease (SCD), Down’s syndrome, and ‎neurofibromatosis-1 ‎[1,2].‎ In a retrospective review, a computed tomography (CT) cerebral angiogram study was ‎conducted on 542 SCD patients to rule in/out MMD, and it showed 23 patients (4%) were diagnosed ‎with MMD [3]. The combination of SCD and MMD confers a much higher risk of ischemic ‎stroke, with SCD-MMD patients having twice the risk of experiencing recurrent ‎cerebrovascular events and being five times more likely to experience recurrent strokes ‎compared to SCD-only patients [4]. ‎

MMD has ‎different etiologies between genetic and environmental factors. Knowledge of these associations between MMD and syndromes is important for practitioners to recognize MMD as a ‎differential diagnosis during the initial assessment‎ [2]‎.‎

The prognosis of MMD relies entirely on an accurate diagnosis and immediate ‎management. Surgical revascularization remains the primary management for MMD; ‎however, an individualized medical treatment plan should be considered based on each ‎patient’s condition [5].‎

## Case presentation

A 32-year-old female, known case of SCD presented to the emergency ‎department by the red crescent, having been found fallen down at home and unresponsive. Upon arrival, she was lethargic, confused, and on oxygen with a non-rebreather mask. Within moments of arrival, she developed ‎generalized tonic-clonic seizure for 10 minutes, which was aborted by diazepam 5 milligram (mg) intravenous (IV). There was no history of fever, neck stiffness, shortness ‎of breath, or seizure in the last few days. In ‎the examination, the patient was afebrile, mildly tachypneic with an oxygen saturation of 88%. A postictal neurological ‎examination found her pupils bilaterally equal and reactive, and she responded to painful ‎stimuli. Her other systems were unremarkable. Drug history: the patient was taking tramadol 50 mg tablet per oral for vaso-‎occlusive crisis attacks.

A computed tomography (CT) brain angiogram scan was ordered. Before the patient was ‎shifted for the CT scan, she developed another generalized tonic-clonic seizure, the gap between the first attack and the second one was five minutes and was ‎aborted by phenytoin 1 gram (gm) IV over 45 minutes. Then the patient developed another seizure attack and was aborted by midazolam 3 mg IV, fentanyl 100 microgram (mcg) IV, and succinylcholine 100 mcg IV, and the patient ‎intubated in the emergency department due to status epilepticus and low Glasgow coma scale 6/15, and she never ‎regained her ‎normal level of consciousness. The intensive care and neurology units were involved to ‎manage her as status epilepticus with acute stroke.

**Figure 1:**
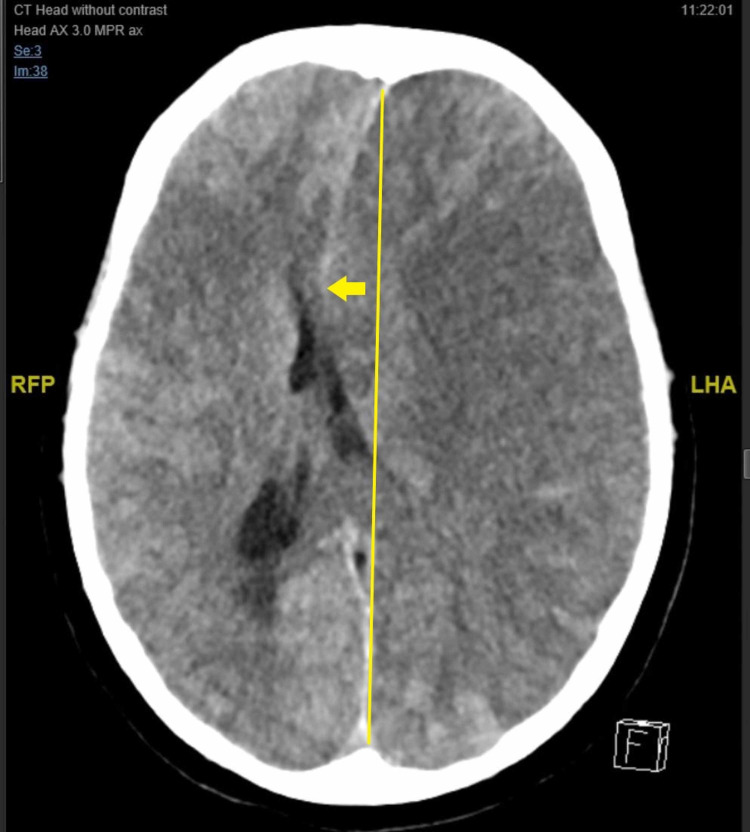
CT brain without contrast - right midline shift

The findings of the CT brain ‎angiogram ‎indicated multiple occlusions in the superior and inferior divisions of the left middle ‎cerebral artery (MCA), and numerous collaterals were detected in the distal branches of the ‎right MCA, which is in keeping with MMD. And newly developed right midline shift was also ‎detected (Figure 1‎). The patient was admitted to the intensive care unit and blood ‎exchange undertaken; however, over the next couple of days, brain death occurred.‎

## Discussion

Moyamoya disease is a rare cerebrovascular disease initially characterized by obliterative ‎vasculopathy followed by compensated proliferative vasculopathy. The name moyamoya ‎means “smoke puff” in Japanese and refers to the presence of small collaterals seen across ‎the basal ganglia and thalamus in response to gradual stenosis and occlusion of the ‎supraclinoid internal carotid artery ‎[1].‎ Moyamoya disease was initially thought to primarily ‎impact Asian people, but it has now been noted globally ‎[2].‎ Incident peaks occur in two ‎age groups: between 5 and 10 years of age in children and between 30 and 50 years of age ‎in adults ‎ ‎[4].‎ The etiopathogenesis is not known, but the syndromic variant can be linked ‎with certain hereditary disorders, such as Down’s syndrome, neurofibromatosis‎-1, and ‎SCD, as encountered by the current patient ‎[6]‎.‎ As the presented patient has different risk factors that give a clue for the main presentation and MMD such as SCD and the age group. 

Clinically, a patient may have ischemic, hemorrhagic, or epileptic conditions. As in our case, the patient presented with refractory status epilepticus that not controlled by first-line and second-line antiepileptic drugs. A few literatures have mentioned atypical ‎symptoms which might associate with MMD presentation, including cognitive dysfunction and behavioral disorders. MMD has a variable ‎presentation related to age group. In comparison to adult-onset, the childhood disease ‎often presents with cerebral infarction, whereas in adults, it often presents with intracranial ‎hemorrhage, arising from the rupture of delicate collateral vessels‎‎ ‎[1,7,8]‎.‎

Epilepsy is a significant manifestation of MMD; however, there are very few reported ‎studies [9]. Ischemic stroke generates a 2.1-fold relative risk of seizure ‎[10]‎. MMD epilepsy is ‎one of the most common forms of post-stroke epilepsy. In MMD, the occlusion of the MCA ‎is often associated with high national institutes health of stroke scale scores and cortical ‎involvement, both of which are identified risk factors for post-stroke epilepsy [10].‎

The primary symptom associated with SCD is acute pain, frequently referred to as a vaso-‎occlusive crisis ‎[11].‎ Opioids, such as tramadol, are used to treat persistent mild to ‎moderate pain related to SCD ‎[12]‎. However, seizures are a major complication of tramadol ‎use, which may occur with therapeutic or toxic doses ‎[13-15].‎ Tramadol‐related seizures are ‎usually controlled with benzodiazepines; however, several studies have demonstrated that ‎benzodiazepines can increase the morbidity and potential lethality of tramadol overdoses, ‎even at therapeutic dose levels ‎[8,16,17].‎ As reported by Shadnia et al., ‎ patients with ‎repeated seizures were given almost twice as much tramadol as patients with single ‎seizures [18]‎.‎

Imaging studies are a crucial step for confirming MMD. Although MRI angiography is used to ‎validate diagnoses and detect vasculopathies, intracranial stenosis indicating Moyamoya ‎can be seen using CT angiography ‎[1]‎. Currently, no evidence suggests that drug therapy will ‎slow or even reverse the development of MMD. Current drug therapy for MMD is confined ‎to clinical symptoms ‎[19], as many studies have demonstrated that surgical ‎revascularization is considered the primary treatment and preventive procedure for MMD. ‎According to a meta-analysis study, the estimated rate of symptomatic progression is 2.6% ‎after surgery ‎[20].‎

## Conclusions

This case highlights the importance of considering MMD as a differential diagnosis when ‎dealing with patients in the third decade of life who have SCD and complain of recurrent ‎seizures that are not aborted by an initial dose of benzodiazepine. It also emphasizes the ‎utilization of cerebral angiography in SCD patients presenting with recurrent seizures to ‎rule out/in MMD. In this case, we emphasize the precaution of considering the side effects ‎of tramadol when managing the acute pain of SCD with MMD, as they might exacerbate ‎seizure conditions, as demonstrated in previous studies.‎
